# Change-of-direction ability in soccer is angle-specific: implications for multi-angle testing and speed deficit calculation

**DOI:** 10.5114/biolsport.2026.162048

**Published:** 2026-05-22

**Authors:** Georgios-Marios Giannakopoulos, Pierros Thomakos, Athanasios Tsoukos, Gregory C. Bogdanis

**Affiliations:** 1 School of Physical Education and Sports Science, National and Kapodistrian University of Athens, Athens, Greece

**Keywords:** Change-of-direction speed deficit (%CODD), Linear sprinting, Performance testing, Agility, Sprinting

## Abstract

The ability to change direction is crucial in soccer due to the high frequency of rapid movement adjustments during match play. This study examined the effects of change-of-direction (COD) angle (45°, 90°, 180°) and limb dominance on sprint speed in 18 experienced male soccer players using a multi-angle fieldbased assessment. Athletes completed a 20 m linear sprint and three 20 m COD sprints, each incorporating a single directional change at 15 m. Mean speed was assessed across the 0–10 m, 10–20 m (COD speed), 10–13 m (entry speed), and 13–20 m segments, and percentage COD speed deficit relative to linear sprinting (%CODD) was calculated. Mean speed in the 0–10 m segment was identical between linear and COD sprints (5.18 ± 0.28 to 5.28 ± 0.28 m · s^−1^). Using the linear 0–10 m speed as a comparator, %CODD values were positive or near-zero for 45° and 90° (+21.0 ± 12.7% and −2.9 ± 7.7%, respectively), indicating greater COD speed than linear, with a negative value only for 180° (−25.2 ± 4.3%). When COD ability was quantified relative to the 10–20 m segment of the linear sprint (7.53 ± 0.37 m · s^−1^), a clear and progressively greater %CODD was observed across angles (45°: −15.6 ± 7.6%; 90°: −32.2 ± 4.7%; 180°: −47.7 ± 2.7%; all p < 0.001). Entry speed declined from 45° to 90° COD (6.55 ± 0.45 to 5.77 ± 0.34 m · s^−1^, p < 0.001), with no difference between 90° and 180° (5.79 ± 0.31 m · s^−1^, p = 1.0). Kendall’s tau correlations showed moderate agreement in %CODD between adjacent angles, but not between 45° and 180°, with no effect of limb dominance (all p > 0.05). In conclusion, %CODD should be calculated using matched linear segments. Angle-dependent and individual variability in %CODD support multi-angle, rather than single-direction, assessment and training.

## INTRODUCTION

Change of direction (COD) is a critical technical skill in many individual and team sports, requiring athletes to rapidly alter their movement path at different angles while maintaining high movement speed [1–4]. During soccer match play, athletes perform approximately 700 CODs in multiple directions, with nearly 300 executed without ball possession and around 100 at angles greater than 90° [5–6]. These COD tasks involve rapid deceleration, a plant-foot push-off, and re-acceleration into a new direction [1, 7–8]. Directional angle strongly influences achievable velocity and mechanical demands, with larger directional changes (i.e., sharper COD angles) requiring greater braking and propulsive forces [9–11]. Accordingly, CODs ≤ 90° are considered velocity-dominant, whereas sharper (i.e., directional changes ≥ 90°) are force-dominant, with maneuver time and demands increasing as angle increases [12–16].

A defining feature of sharper CODs is a reduction in approach and execution speed [15]. Compared with 45° CODs, athletes typically perform 90° CODs at lower velocities and with greater deceleration, reflecting increased braking demands [9, 14, 17]. Speed during COD is therefore a key determinant of performance, particularly at sharper angles where efficient deceleration and re-acceleration are critical to match specific demands such as pressing the opposition players and regaining possession through ball recovery and tackling [9, 14–15]. Although numerous COD tests exist, such as the 5-0-5 and the T-test [18–19], most assess a single angle, and it remains unclear whether COD ability and speed-related characteristics are consistent across angles. Moreover, changes in running speed during COD at different angles have not been comprehensively examined [18–20].

To better isolate COD ability from linear sprint performance, the change of direction deficit (CODD) has been proposed as the difference in time or velocity between linear sprinting and COD performance over the same distance [18, 20–21]. In tests such as the 5-0-5, CODD has traditionally been calculated using a straight 10 m sprint, often derived from a 0–10 m split [18–19]. However, this does not fully reflect the rolling entry used in the timed portion of the test. Consequently, some studies have proposed using a flying 10 m split (e.g., 10–20 m of a 20 m sprint) as a more contextspecific linear comparator [22–23, 24]. Notably, although sprint speed contributes to COD performance [25], faster athletes often display larger CODD values due to greater deceleration demands and inertia management requirements [13, 26–30].

The influence of leg dominance further complicates COD assessment, with inconsistent and angle-specific findings reported [15–16, 31–32]. While superior performance has been reported with the preferred limb during side-step cutting [33], other studies show minimal or no effects in standardized COD tests, including 5-0-5 and 90° protocols [31–32, 34–35]. These discrepancies suggest that limb dominance effects depend on COD angle, task demands, and the performance metrics used.

Despite the importance of multidirectional COD ability in team sports, relatively few field-based studies have examined the effects of COD angle on speed and CODD [16, 32–33, 36], and even fewer have used distance-matched protocols allowing fair comparisons across angles [16, 32–33]. Therefore, the primary aim of this study was to examine how COD angle influences speed-related performance variables in soccer players. A secondary aim was to determine whether COD ability is angle- or limb-specific and whether performance remains consistent across different COD angles. We hypothesized that sharper COD angles would result in greater reductions in mean speed and higher COD deficits, and that COD performance would vary between angles due to their distinct mechanical demands.

## MATERIALS AND METHODS

### Study design

This study used an experimental repeated-measures design to examine the effects of COD angle and limb dominance on speed-related performance variables in soccer players. A priori power analysis (G*Power software, version 3.1.9.7) based on previous research (Cohen’s *d* = 1.56–8.96) [15], showed that at least six individuals were required to provide a conservative effect size of *f* = 0.5 (equivalent to *d* ≈ 1.0) with *α* = 0.05, power = 0.80 and correlation among repeated measures *r* = 0.50. Given the experimental study design, a sample of 18 male soccer players was recruited to ensure that the study was adequately powered to detect effects of the expected magnitude. To explore the effects of COD angle and leg dominance on sprint speed and percentage change of direction deficit (%CODD), a repeated measures design was employed with one group of participants and six repeated measurements (2 legs, i.e. dominant and non-dominant × 3 COD angles). Participants completed two testing sessions, separated by one week and scheduled at least 72–96 h after match play. The order of testing conditions was randomized and counterbalanced using a simple randomization procedure (www.randomizer.org). Testing involved a linear 20 m sprint and familiarization with the multi-angle COD test on the first day, while on the other day participants performed the 20 m multi-angle COD tests at three different angles (45°, 90°, 180°) with both the dominant and non-dominant leg.

### Subjects

Eighteen experienced soccer players (age: 25.0 ± 6.6 years; training experience: > 12 years, height: 177 ± 0.04 cm; body mass: 75.5 ± 8.3 kg) were recruited. Prior to participation, volunteers were fully informed about the study protocol, potential risks, and their rights, including the freedom to withdraw at any time. Then, written informed consent was obtained. The research protocol was approved by the Institutional Review Board of the School of P.E. and Sport Science of the National and Kapodistrian University of Athens (Approval no. 1799/22-5-2025) and conducted in accordance with the ethical principles outlined in the Declaration of Helsinki of 1964 as revised by the World Medical Association in 2024.

### Preliminary measurements and warm-up

To determine the dominant leg, players were asked to indicate the leg they preferred to use for shooting, passing, and controlling the ball [37]. The participants were familiar with the testing procedures, as their regular training included COD and maximal sprinting drills. Testing was performed on a synthetic grass soccer court prior to the daily training session. Players wore standard training attire and soccer-specific shoes. All procedures were supervised by the team’s technical staff and sport scientists. Prior to testing, participants completed a standardized warm-up consisting of 5 minutes of light, self-paced aerobic activity, followed by static and dynamic stretching of the lower limbs, as well as progressive sprints and changes of direction. The warm-up lasted approximately 10 minutes. A passive recovery period of 5 minutes was allowed between the warm-up and the beginning of testing. Immediately afterward, participants received verbal instructions and performed two familiarization trials at a moderate pace.

### Measures

Athletes completed a 20 m linear sprint and, on a separate day, three 20 m COD sprints, each incorporating a single directional change with the dominant and non-dominant leg at three angles of 45°, 90° and 180° (see Figure 1). Each subject was instructed to run as fast as possible for 20 m until the finish line. Two trials were performed at each angle and leg, with 3 minutes of passive recovery intervals (12 sprints in a randomized and counterbalanced order), and the average of the two trials per angle was taken for further analysis. For the 20 m linear sprint, the best splits (0–10 m, 10–20 m) were obtained [23]. Performance time was measured with photocells (Microgate Witty SEM System Timing Gates, Bolzano, Italy) positioned as shown in Figure 1. Participants started each trial using their to exit speed. On the left, the 20 m linear sprint test is shown. preferred leg, with the foot positioned 0.4 m behind the first timing gate. The timing gates were set at a height of 0.6 m and at 1.5 m apart. Mean speed was calculated for the initial 10 m segment (0–10 m or AB), the 3 m segment (10–13 m or BC) corresponding to the entry speed, and for the remaining 7 m until the finish line (13–20 m or CD) which included CODs at 15 m for all angles (45°, 90°, 180°). For the 180° COD this 7 m segment was the CB distance, including a CC segment of 2+2 = 4 m and an additional CB segment of 3 m accounting for exit speed was recorded.

**FIG. 1:**
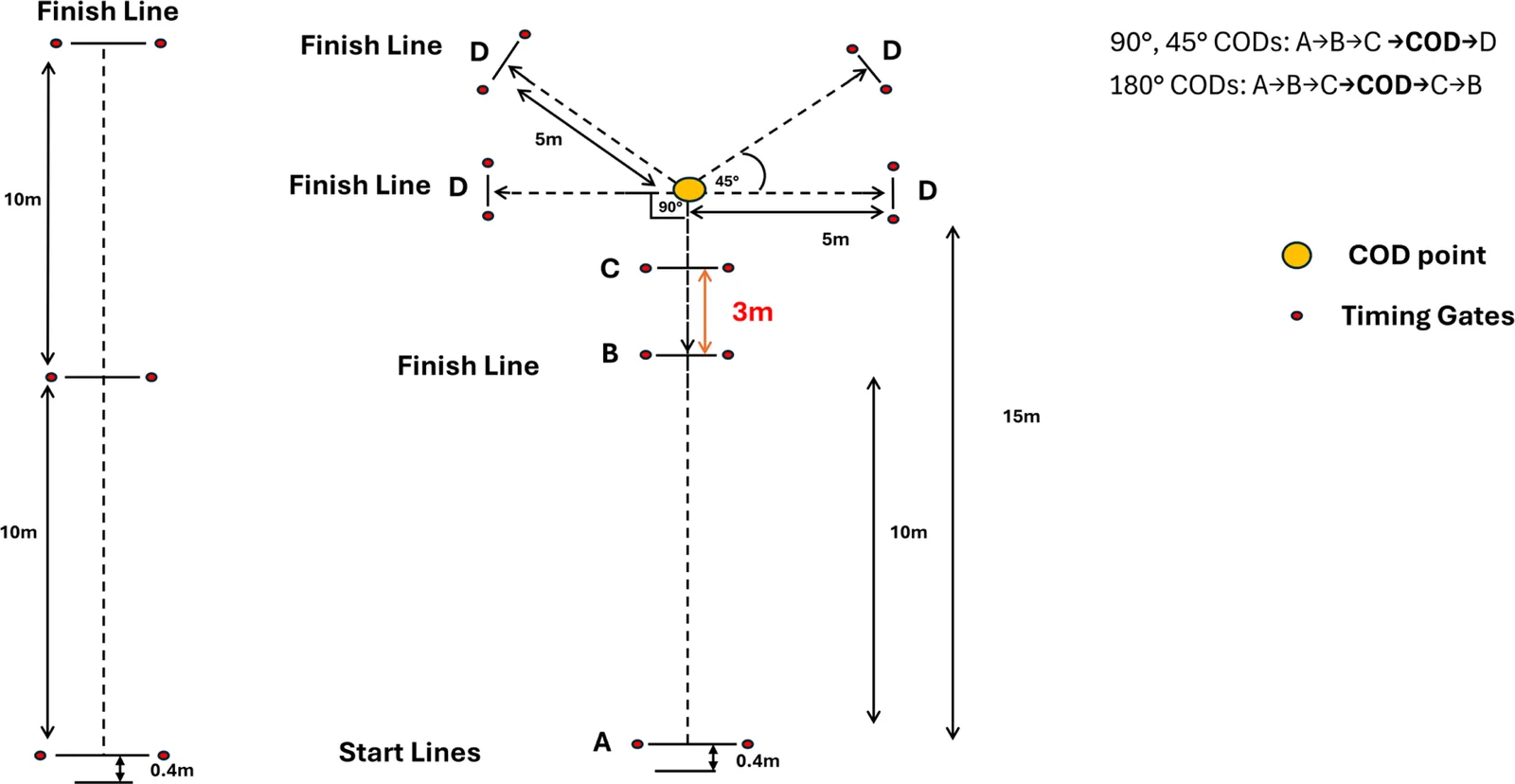
The multi-angle COD test. Segment A-B (0–10 m) corresponding to initial speed, segment BC (10–13 m) or entry speed, and segment CD (13–17 m) at 45° and 90° with COD at 15 m. At 180° the trial involved C-C or 2+2 = 4 m and C-B corresponding to exit speed. On the left, the 20 m linear sprint test is shown.

### CODD calculations

The percentage of speed loss or COD deficit (%CODD) [21], was calculated using the formula:

$$\%CODD=\frac{\left(Ucod-Ulinear\right)}{Ulinear}\times 100\%$$

where Ucod is the mean speed for the 10–20 m, i.e. the BD segment for 45°, 90° or the BB segment for 180° CODs. For the U_linear_ segment, we used either the mean speed at the first 10 m segment of the linear sprint (0–10 m) [18–19, 38], or the second 10 m segment of the linear sprint 10–20 m, as used in more recent studies [22–24], where U_linear_ is the mean speed of the second (10–20 m) linear segment.

### Statistical analyses

All statistical analyses were conducted using JASP Statistics (0.95.4.0). All variables are presented as means ± standard deviations. A two-way repeated measures analysis of variance (ANOVA) with two factors (angles × leg), 3 levels of angle (45°, 90°, 180°) and 2 levels of leg (dominant, non-dominant) was performed for all dependent variables. A Greenhouse–Geisser correction was used when the assumption of sphericity was violated. Additionally, one-way repeated measures ANOVAs with 7 levels (3 angles-2 Legs = 6 conditions of COD test+1^st^ or 2^nd^ 10 m linear) were conducted to examine differences between the first 10 m of the multi-angle COD test trials and the first 10 m of the 20 m linear test and also to compare the second 10 m segment of the multi COD tests (segment BD for the 45° and 90°, and segment B to B for the 180° COD test) and the second 10 m (10–20 m) of the linear sprint test. Measurement reliability was evaluated using the average-measures intraclass correlation coefficient (ICC).

When significant main effects or interactions were observed, Bonferroni post hoc tests were applied. Effect sizes for pairwise comparisons were estimated using Hedges’ *g* [39] and interpreted as small (< 0.3), medium (0.3–0.8), or large (> 0.8). Effect sizes for main effects and interactions were assessed using partial eta squared (*η*^2^*p)* [40], and interpreted as small (0.01–0.06), moderate (> 0.06–0.14), or large (> 0.14). The level of statistical significance was set at *α* = 0.05 for all analyses. In addition, standardized zscores were calculated for % CODD across angles (45°, 90°, 180°) and limbs (dominant and non-dominant), and then each score was transformed to a ranking of 1 (highest score) to 18 (lowest score), for the 18 participants. Then, Kendall’s tau *b* correlations in ranking scores were used to examine whether athletes maintained similar relative performance rankings in % CODD across angles (45°, 90°, 180°) for both the dominant and non-dominant limb. The level of agreement was interpreted as poor (< 0.20), fair (0.21–0.40), moderate (0.41–0.60), good (0.61–0.80) and strong (0.81–1.00) [41–42].

## RESULTS

Based on average measures ICC, all variables demonstrated good-to-excellent reliability for both the dominant and non-dominant leg (ICC = 0.71–0.97).The two-way ANOVA and Post-hoc tests, for linear mean speed for the 0–10 m segment revealed a non-significant angle × leg interaction (*F*_(2, 34)_ = 0.001, *p* = 0.999) and also nonsignificant main effects of angle and leg (*F*_(2, 34)_ = 1.889, *η*^2^*p* = 0.100 and *F*_(1, 17)_ = 2.058, *η*^2^*p* = *0.108, p* > 0.05, g = 0.09–0.2, see Figure 2a). Furthermore, the result of the one-way ANOVA (*F*_(6, 102)_ = 1.235, *p* = 0.295) did not show any differences between the first 10 m mean speed of the multi-angle COD test (5.26 ± 0.30, 5.22 ± 0.34, 5.19 ± 0.26 m · s^−1^, for 45°, 90° and 180° respectively, averaged for both legs) and first 10 m of the 20 m linear test (5.26 ± 0.26 m · s^−1^).

**FIG. 2:**
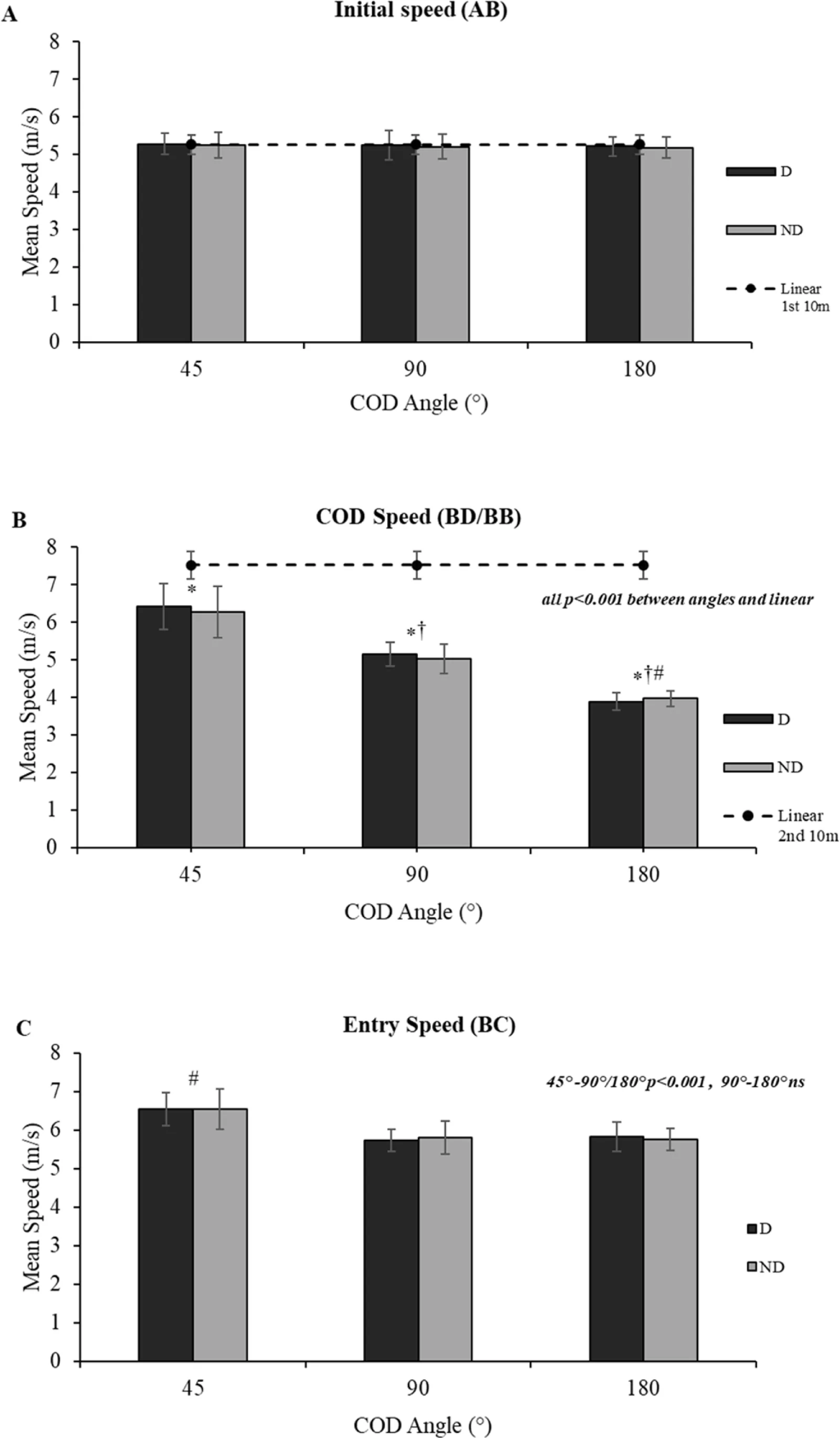
Mean speed for three different segments of the 20 m change of direction sprint. Panel A refers to the initial (0–10 m) or segment AB; panel B refers to the change of direction segment (10–20 m or segment B-D for 45,90° and segment BB for 180°); panel C shows the entry speed (10–13 m or segment BC). Dotted line in panel Α shows the mean speed for the first 10 m of the linear sprint (0–10 m). Dotted line in panel B shows the mean speed for the second 10 m of the linear sprint (10–20 m). *: p < 0.001 from reference speed; †: p < 0.001 from 45°; #: p < 0.001 from 90° and 180°.

Mean speed in the second 10 m segment of the COD tests (segment BD for 45° and 90° and segment BB for the 180° COD test) is shown in Figure 2b. A significant main effect of Angle, *F*_(1.198, 20.369)_ = 287.87, *p < 0.001, η*^2^*p* = 0.944 was found, with no significant main effect of Leg, (*F*_(1, 17)_ = 1.068, *p* = 0.316, *η*^2^*p* = 0.059,) and non-significant angle × leg interaction (*F*_(2, 34)_ = 3.996, *p* = 0.028, *η*^2^*p* = 0.190). Post hoc comparisons showed that mean speed was significantly higher for 45° compared to 90° (6.42 ± 0.62 / 6.28 ± 0.68 vs. 5.15 ± 0.32 / 5.03 ± 0.40 m · s^−1^, *p* < 0.001, *g* = 2.69), while both were faster than mean speed at 180° (3.89 ± 0.23 / 3.97 ± 0.21 m · s^−1^, *p* < 0.001, *g* = 2.49 and 5.18) with no differences between legs (g = 0.128). The one-way ANOVA and posthoc tests revealed significant differences between the 10–20 m segment of the linear test and the 10–20 m segment of all COD tests (7.53 ± 0.37 m · s^−1^, *F*_(2.576, 43.796)_ = 297, *p* < 0.001, *η*^2^*p* = 0.946 *g* = 2.43 to 7.98 (Figure 2b).

The mean speed for the 3 m BC segment (entry speed) for the three COD tests is shown in Figure 2c. A significant main effect of Angle, *F*_(1.512, 25.701)_ = 37.796, *p* < 0.001, *η*^2^*p* = 0.690 was revealed, without any significant main effect for leg (*F*_(1, 17)_ = 4.416 × 10^−4^, *p* = 0.983) or angle × leg interaction: *F*_(2, 34)_ = 1.067, *p* = 0.355, *η*^2^*p* = 0.059). Post hoc pairwise comparisons showed that entry speed was higher at 45° compared to 90° and 180° (6.55 ± 0.45 vs. 5.77 ± 0.34 vs. 5.79 ± 0.31 m · s^−1^, *p* < 0.001, *g* = 1.89 and 1.82), with no difference between 90° and 180° (*p* = 1.0, g = 0.06) and no difference between legs (g = 0.001).

Figure 3 shows the mean and individual percentage changes in %CODD averaged over the level of leg, since there was no difference between legs. When the %CODD was calculated relative to the 10–20 m linear speed (%CODD, Figure 3a), the ANOVA showed a significant main effect of angle, (*F*_(1.253, 21.302)_ = 319.063, *p* < 0.001, *η*^2^*p* = 0.949), and significant angle x leg interaction (*F*_(2, 34)_ = 4.079, *p* = 0.026, *η*^2^*p* = 0.194) with no leg effects (*F*_(1, 17)_ = 1.288, *p* > 0.27, *η*^2^*p* = 0.070). Post hoc comparisons showed that %CODD was significantly lower at 45° and increased with COD angle (−15.6 ± 7.6, −32.2 ± 4.7% and −47.7 ± 2.7%), for 45°, 90° and 180°, respectively, (*g* = 2.6 to 5.2 all *p* < 0.001) but non-significant difference between legs (*g* = 0.142).

**FIG. 3:**
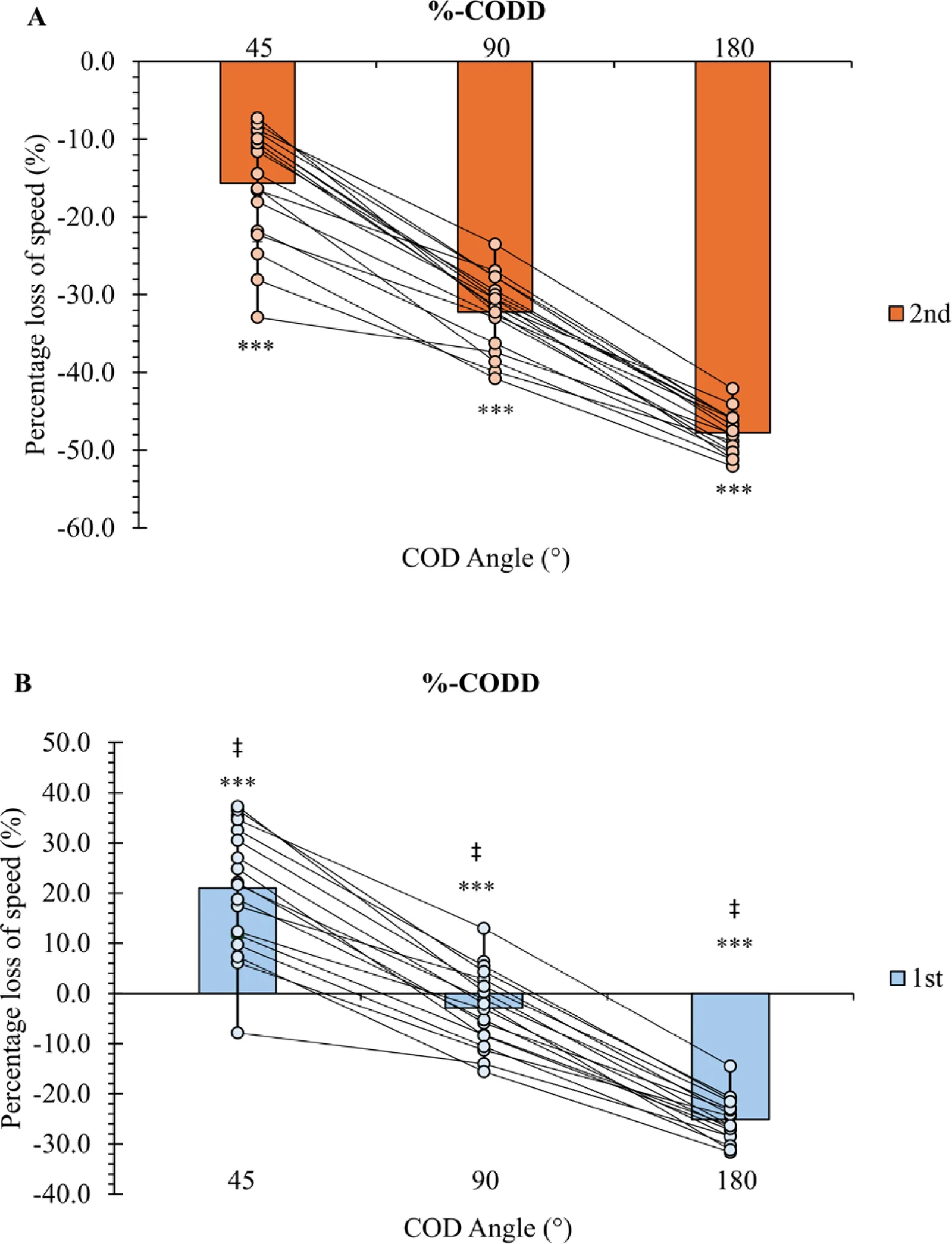
Mean and individual data points of percentage change of direction speed deficit (BD-45,90° and BB -180° equal to 5+5 m), compared to the second 10 m speed (10–20 m, panel a.) or the first 10 m speed, panel b) (0–10 m, panel across angles (45°, 90°, 180°). ***: p < 0.001 between all angles; ‡: p < 0.001 between corresponding angles of panel A and panel B.

When the %CODD was calculated relative to the initial (0–10 m) linear speed (Figure 3b), the ANOVA showed a significant main effect of angle (*F*_(1.223, 20.791)_ = 287.685, *p* < 0.001, *η*^2^*p* = 0.944), and angle x leg interaction (*F*_(2, 34)_ = 3.988, *p* = 0.028, *η*^2^*p* = 0.190). No significant interaction effect between COD angle and limb was observed (*F*_(1, 17)_ = 1.222, p = 0.28), indicating that the influence of angle on performance did not differ between limbs. Post hoc comparisons showed that %CODD showed a “deficit” only for the 180° trial (−25.2 ± 4.3%), while for the 90° the deficit was small (−2.9 ± 7.7%) and for the 45° trial, there was a positive value for “deficit”, indicating higher COD speed (+21.0 ± 12.7%, Figure 3), (*g* = 2.25 to 4.66 all *p* < 0.001) but non-significant difference between legs (*g* = 0.12).

Figure 4 presents individual values for percentage changes in mean speed across the segments of the test at each angle, averaged for the two legs, as there was no significant difference between dominant and non-dominant leg. The individual data illustrate speed changes from the AB segment (initial speed or 0–10 m) to the BC segment (entry speed or 10–13 m) and then to the CD segment at 45° and 90°, and CB at 180° (13–20 m) including the COD (see Fig 1).

**FIG. 4:**
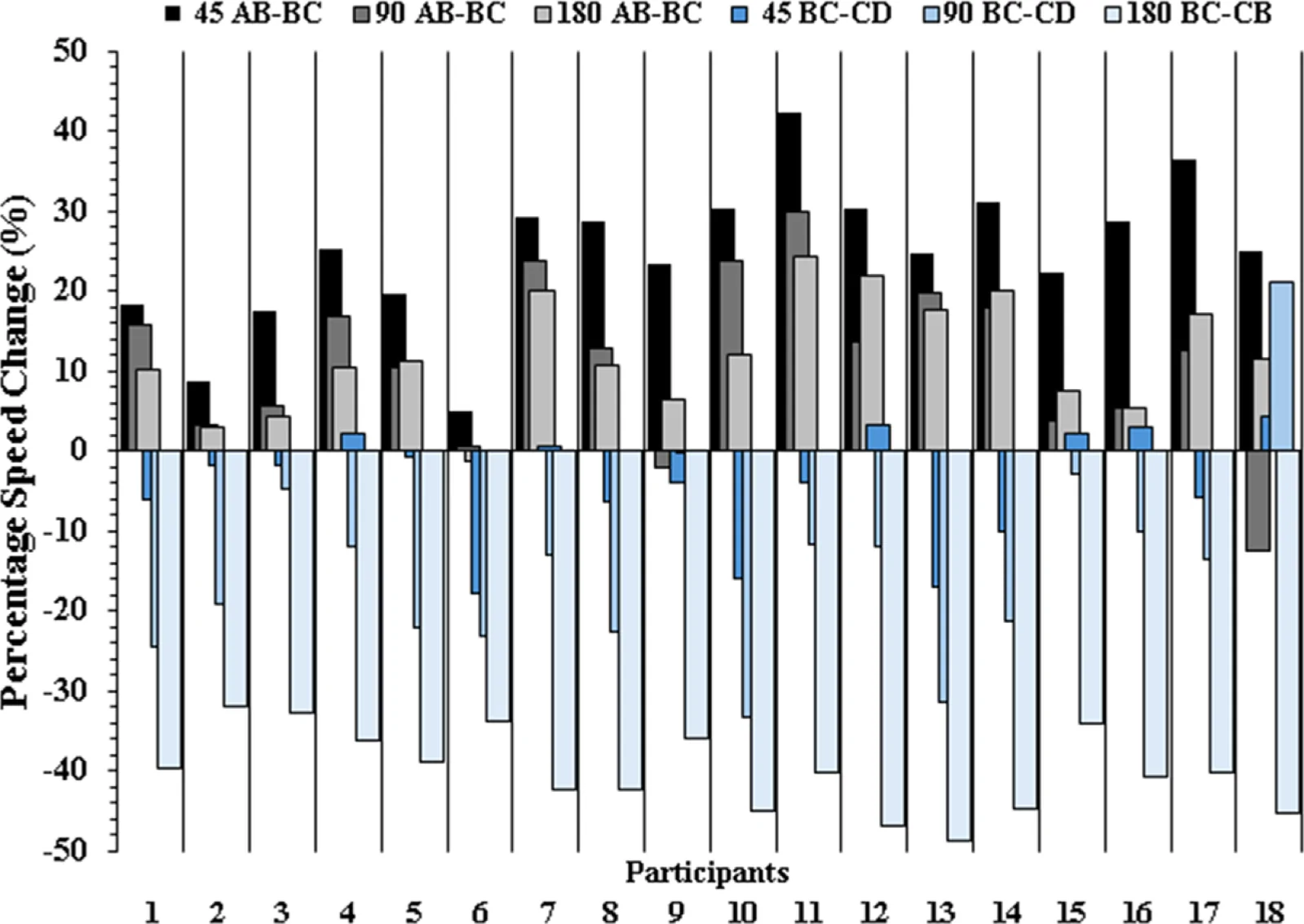
Percentage changes in mean speed across the segments (AB-BC, BC-CD at 45° and 90° and BC-CB at 180°) of the multi angle COD test at a): 45° change of direction (COD), b): 90° COD and c): 180° COD. Positive values indicate speed increase and negative values speed decrease.

To evaluate individual performance profiles regarding the consistency of %CODD across angles, we compared the relative ranking of z-scores. Kendall’s tau correlations revealed that for the dominant leg, a moderate relationship was observed between 45° and 90° (*τ* = 0.556, *p* < 0.001), and between 90° and 180° (*τ* = 0.477, *p* = 0.005), whereas the association between 45° and 180° was non-significant (*τ* = 0.268, *p* = 0.131). Similarly, for the non-dominant leg a moderate association was revealed between 45° and 90° (*τ* = 0.542, *p* = 0.001), and 90°–180° (*τ* = 0.412, *p* = 0.017). However, the association between 45° and 180° rankings was also non-significant (*τ* = 0.268, *p* = 0.131). Dominant and non-dominant legs showed fair to moderate agreement at the same angle (45°: *τ* = 0.516 *p* = 0.002; 90°: *τ* = 0.346, *p* = 0.048; 180°: *τ* = 0.438, *p* = 0.011).

## DISCUSSION

This study examined the effects of COD angle and limb dominance on speed-related variables in experienced soccer players using a multiangle field-based COD assessment. The primary finding was that the method used to calculate COD deficit fundamentally alters the interpretation of COD performance, particularly at angles < 180°. When COD ability was quantified relative to the second 10 m of the linear sprint, a clear and progressive speed deficit was consistently observed across all angles, reaching almost ~50% at 180°. In contrast, using the first 10 m as a comparator, as commonly applied in traditional tests such as the 5-0-5 [18], produced misleading outcomes at 45° and 90°, including near-zero or positive percentage change of direction deficit values. A second key finding was that COD performance was angle-specific but not limb-specific, with individuals who ranked highly at one angle not necessarily performing well at other.

The progressive increase in %CODD with greater COD angles reflects the escalating mechanical and neuromuscular demands imposed by sharper directional changes. As COD angle increases, athletes must dissipate and reorient a larger proportion of horizontal momentum, requiring greater braking impulse, longer ground contact times, and higher propulsive forces during re-acceleration [9–10, 13, 43]. Consequently, mean speed during COD execution decreases despite unchanged linear sprint capability, resulting in larger relative speed deficits. In the present study, %CODD increased from modest losses at 45° to substantial reductions at 90°, reaching almost ~50% at 180°, values comparable to those reported in other team-sport athletes [16, 32, 36]. This nonlinear increase indicates that sharper CODs represent qualitatively different movement challenges, shifting from velocity-dominant to force-dominant demands [32], highlighting the importance of multi-angle COD assessment.

Kendall’s tau analyses further demonstrated that COD performance is highly angle-specific. Moderate correlations were observed only between adjacent angles (45°–90° and 90°–180°), whereas the association between 45° and 180° was fair and non-significant, indicating limited performance transfer when task demands differ substantially. Shallow CODs (< 90°) are predominantly velocity-oriented, whereas sharper CODs (> 90°) impose greater braking, force production, and neuromuscular control demands [32, 43]. As a result, athletes performing well at lower angles may struggle to maintain relative performance at sharper angles. These findings confirm that COD ability represents a spectrum of task-specific qualities rather than a single transferable capacity, supporting the inclusion of multiple angles in both testing and training. In relation to real game scenarios, this may have practical implications, as players with lower performance at sharper angles may be less effective during actions requiring rapid braking and re-acceleration, such as defensive recovery, pressing, tight marking, or quick directional adjustments when contesting possession [5, 44, 45].

Entry speed also influenced COD performance, with athletes approaching 45° CODs at higher velocities than 90° and 180°. This aligns with previous findings showing that athletes self-regulate approach speed in anticipation of braking demands, entering sharper CODs at lower velocities to maintain control and mechanical safety [9–10, 15]. Shallow-angle CODs allow greater velocity preservation, whereas sharper angles require greater deceleration prior to push-off [8]. Accordingly, reduced entry speeds at 90° and 180° likely reflect anticipatory strategies to manage increased braking and re-acceleration demands.

Notably, entry speed did not differ between the 90° and 180° CODs, suggesting that athletes may perceive these tasks as similarly demanding. Although total momentum redirection is greater at 180°, both tasks require substantial braking and force reorientation, potentially exceeding a threshold beyond which athletes adopt comparable conservative strategies [9, 32, 43]. However, this similarity may also reflect a methodological limitation, as entry speed was assessed 2 m before the COD point, potentially masking late-phase deceleration during the 180° task where braking demands peak [1, 13, 17]. Continuous speed tracking or closer gate placement may better capture these effects in future studies.

Figure 4 further illustrates marked inter-individual variability in speed responses across COD angles and task phases. Participants differed substantially in approach-speed regulation and in the magnitude of speed loss during COD execution, with reductions during the COD phase consistently exceeding those observed during approach. Importantly, these patterns were not uniform across angles, as individuals who managed speed effectively at one angle often exhibited greater losses at another. This reinforces the highly individual and angle-dependent nature of COD performance and supports the need for multi-angle assessment and individualized interpretation.

The comparison between the two %CODD calculation methods represents a key methodological insight. When COD deficit was calculated relative to the second 10 m of the linear sprint, %CODD increased progressively with angle, reaching almost ~50% at 180°, values closely aligned with time-derived CODD reported in basketball players (43–51%) [16]. However, deficits at 45° and 90° were two- to five-fold greater than previously reported [16], likely reflecting differences in test design, approach distance, and sport-specific movement strategies. Soccer players may approach CODs at higher velocities, particularly at shallower angles, amplifying braking demands and relative speed loss.

In contrast, calculating %CODD relative to the first 10 m of linear sprinting, as used in traditional assessments such as the 5-0-5 [18], produced distorted interpretations, particularly for CODs < 180°. Because this segment is acceleration-dominant and characterized by lower velocities, its use artificially reduces computed deficits, resulting in near-zero or paradoxical positive values. These findings demonstrate that acceleration-based reference speeds are inappropriate for evaluating COD tasks performed at higher velocities and strongly support the use of velocity-matched linear sprint segments.

Despite the current findings, some limitations should be acknowledged. Speed was assessed as mean velocity rather than continuously, limiting insight into instantaneous deceleration and re-acceleration strategies. Entry speed was measured 2 m before the COD point, which may have masked late-phase deceleration, particularly during sharper CODs such as the 180° task. The absence of kinetic and kinematic data restricts interpretation of the mechanical factors underpinning angle-specific COD performance. Additionally, the a-priori power analysis was based on detecting large effect sizes; therefore, smaller but potentially meaningful effects may not have been detected, increasing the possibility of Type II error. Nevertheless, this field-based multi-angle COD assessment provides ecologically valid information for applied practice. As the sample comprised experienced male soccer players, caution is warranted when generalizing these findings to other populations or sports.

## CONCLUSIONS

This study showed that COD performance is strongly influenced by directional angle and is highly sensitive to the method used to quantify performance loss. Velocity-matched reference speeds consistently revealed meaningful deficits across all angles, whereas acceleration-based comparators produced misleading outcomes for CODs < 180°. Performance rankings confirmed that COD ability is angle specific rather than a single transferable quality, while entry-speed regulation suggested similar perceptual demands for 90° and 180° CODs. No limb differences were observed, indicating bilateral consistency within this sample of soccer players. Collectively, these findings support the use of multi-angle COD assessments and velocity-appropriate reference speeds to accurately characterize performance and guide targeted training interventions. Shallower angles (≤ 90°) are typically more velocity-oriented and commonly occur during high-speed actions in soccer such as pressing, tracking opponents, or adjusting running lines in transition. Therefore, training may benefit from drills emphasizing high-speed directional adjustments and reactive agility tasks. In contrast, sharper angles (≥ 90°) which often occur during defensive recovery, marking, or tight-space maneuvers around opponents, require greater braking and propulsive force production and may therefore benefit from exercises focusing on deceleration capacity, eccentric strength, and rapid reacceleration.
